# Primary Iliac Venous Leiomyosarcoma: A Rare Cause of Deep Vein Thrombosis in a Young Patient

**DOI:** 10.1155/2011/123041

**Published:** 2011-06-16

**Authors:** Nelson Oliveira, Emanuel Dias, Ricardo Lima, Fernando Oliveira, Isabel Cássio

**Affiliations:** Department of Angiology and Vascular Surgery, Hospital do Divino Espírito Santo, 9500 Ponta Delgada, Portugal

## Abstract

*Introduction*. Primary venous tumours are a rare cause of deep vein thrombosis. The authors present a case where the definitive diagnosis was delayed by inconclusive complementary imaging. *Clinical Case*. A thirty-seven-year-old female presented with an iliofemoral venous thrombosis of the right lower limb. The patient had presented with an episode of femoral-popliteal vein thrombosis five months before and was currently under anticoagulation. *Phlegmasia alba dolens* installed progressively, as thrombus rapidly extended to the inferior vena cava despite systemic thrombolysis and anticoagulation. Diagnostic imaging failed to identify the underlying aetiology of the deep vein thrombosis. The definitive diagnosis of primary venous leiomyosarcoma was reached by a subcutaneous abdominal wall nodule biopsy. *Conclusion*. Primary venous leiomyosarcoma of the iliac vein is a rare cause of deep vein thrombosis, which must be considered in young patients with recurrent or refractory to treatment deep vein thrombosis.

## 1. Introduction

Deep vein thrombosis (DVT) is diagnosed in 56 to 122 per 100 000 individuals each year [1] and gradually becomes more frequent as age increases [2]. With multiple risk factors recognised, including genetic and exogenous factors [3], DVT's pathogenesis is usually related to venous stasis (obstructive or nonobstructive), disruption of the vascular wall or to hypercoagulable states [3].

Occult tumours may present as DVT. Among these, primary venous leiomyosarcoma remains a rare tumour, with approximately 400 cases published [4] since its first description in 1871 by Perl [5]. However, leiomyosarcoma is the most frequent tumour of the venous system, arising in more than half of the cases in the inferior vena cava (IVC) [6].

Tumours with a venous origin are generally malignant and difficult to diagnose [7] with up to one third of the diagnosis reached *post mortem* [6].

A case of primary venous leiomyosarcoma of the right iliac vein which presented as a recurrent DVT is reported. 

## 2. Clinical Case

A 37-year-old caucasian female presented with pain and oedema of the right lower limb with three days of evolution. Symptoms had started two days after a two-hour flight. Accompanying symptoms were nonquantified fever and dysuria. The patient denied any loss of weight, anoraexia, asthenia, abdominal or lumbar pain, or history of trauma of the right lower limb.

About five months prior to this episode, the patient had been diagnosed with a femoral-popliteal vein thrombosis in the same lower limb and was started on anticoagulation with warfarin, with a therapeutic international normalized ratio (INR) gold of 2-3. Diagnostic testing prior to anticoagulation revealed normal prothrombin time (PT) and activated partial thromboplastin time (aPTT) values, and prothrombotic conditions screening (including lupus anticoagulant, anti-cardiolipin antibodies, antithrombin III, Protein C, Protein S functional and antigenic assays, Leiden V factor, and homocysteinemia) was negative. 

Remaining medical history was notable for depressive syndrome and allergic rhinitis. Along with warfarin, the patient was medicated with mexazolam 1 mg id, Daflon 500 (r) bid, and fluoxetine 20 mg id. The patient was a mother of two healthy children, aged three and twelve years, born from normal full-term deliveries. A previous history of oral contraceptives was found but had been suspended over three years. Metrorrhagia or amenorrhea was denied. Smoking habits of about one pack a day had ceased ten years before the beginning of the present illness. 

In regard to family history, a heavy history of malignancies was found, including a brother who died at age thirty-nine of a pulmonary adenocarcinoma. Among the patient's uncles and grandparents, several cases of cancer (a gastric cancer, a multiple myeloma, a cervical uterine cancer, and a case of prostate cancer), type 2 diabetes mellitus, coronary disease, and cerebrovascular disease were identified. 

Presenting physical examination revealed a fever of 38.2°C and a body mass index of 30.3. Oedema was observed involving the full extension of the right lower limb without cyanosis. All pulses were palpable, symmetrical, and had normal amplitude. Abdominal palpation did not reveal the presence of masses or organomegalies. The remaining physical examination did not identify any other pathological finding. 

A haemoglobin of 12.4 g/dL, an INR of 2.08 and an aPTT of 30.5 seconds were obtained in a laboratory workup. White blood count and differential were normal, reactive C protein was 0.74 mg/dL. Microscopic urinary sediment examination was positive for leucocytes.

Duplex and colour Doppler imaging of the lower limb confirmed a right venous iliofemoral thrombosis with extension to the ipsilateral great saphenous vein and abdominal wall collaterals. Arterial examination was normal.

Initial approach coursed with suspension of oral anticoagulation and systemic thrombolysis with reteplase (rt-PA) in a dose of 9 mg/kg, (10% in a bolus during the first minute followed by a continuous perfusion during one hour of the remaining) followed by a nonfractioned heparin perfusion over 48 hours. Warfarin was resumed, and therapeutic low-molecular-weight-heparin was maintained until achievement of an INR level of 2-3.

A contrast-enhanced computerized tomography scan (CT-scan) of the abdomen and pelvis revealed extension of right iliac vein thrombosis to the IVC and also noted an “exuberant agglomerate of thrombosed venous collaterals” in the right lower quadrant of the abdominal wall, according to the report. No masses were mentioned.

Positive hemocultures were obtained for *Serratia Marcescens*, sensitive to antibiotic therapy in course (Piperacilin and tazobactam). Uroculture was negative.

Antinuclear autoantibodies (anti-Sm; anti-Ro/SSA; anti-La/SSB; antitopoisomerase [Scl-70]; anticentromere and anti-t-RNA-sintetase [Jo1]) and serologic tumour markers (Carcinoembryonic antigen, Cancer antigen 125, Cancer antigen 15.3, and Cancer antigen 19.9) were negative.

The patient was discharged after 19 days of hospitalization under oral anticoagulation with progressive symptomatic relief.

A month after discharge, the patient was readmitted with a recurrence of pain and oedema of the right lower limb. Despite oral anticoagulation, oedema hada extended to the left lower limb, lumbar region, and abdominal wall (right lower quadrant), and a painful ill-defined subcutaneous nodule with an approximate diameter of 4 centimetres was now palpable in the same abdominal quadrant. Pulses were not palpable in the right lower limb due to extensive oedema, but arterial duplex and colour Doppler remained unchanged. A mild normocytic normochromic anaemia was now found on laboratory workup although acute visible losses were not identified.

**Figure 1:**
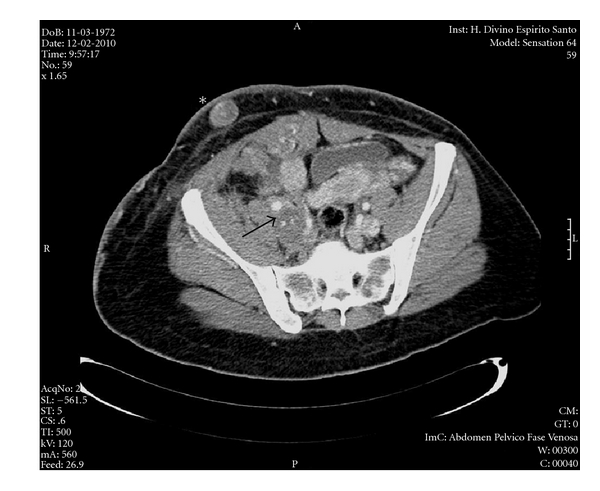
Abdominal and pelvic CT scan. A mass (arrow) was described as an “agglomerate of venous structures” involving a dilated right iliofemoral axis, but its tumor aetiology was not recognised. A nodule is observed but was reported as “thrombosed collateral circulation in the subcutaneous adipose plane of the ipsilateral inferior quadrant of the abdominal wall” (∗). The definitive diagnosis of primary venous leiomyosarcoma was obtained from a biopsy of the nodule.

An abdominal and pelvic contrast-enhanced CT scan revealed thrombus progression to the suprahepatic IVC. The presence of tumour was not reported although an increase in maximum diameter of “the thrombosed collateral circulation” of the pelvis and abdominal wall was noted (Figure 1). An abdominal magnetic resonance (MRI) failed to further clarify this finding.

Screening for warfarin resistance did not identify polymorphisms of CYP2C9 and VKORC1 genes. Other thrombogenic genetic polymorphisms were tested including: F5 (G1691A), FII (G20210A), MTHFR (C677T e A1298C), and PAI-1 (4G/5G). An MTHFR C677T-CT variant of the gene was identified. Although associated with an increased level of homocysteinemia in carriers, an allelic frequency of 41.68% has been reported in our resident population [8].

The abdominal wall nodule biopsy revealed a pleomorphic leiomyosarcoma of vascular origin. Fusiform and multinucleated cells predominated, with a high rate of atypical mitotic figures (more than 50 per 10 amplified fields). This nodule was positive for smooth cell immunohistochemical markers (calponin, desmin, HHF35, alfa-actin, smooth muscle actin, and h-caldesmon) and was negative for epithelial markers (keratin), neuronal markers (S100) hematopoietic markers (CD34), oestrogen, or progesterone receptors.

**Figure 2:**
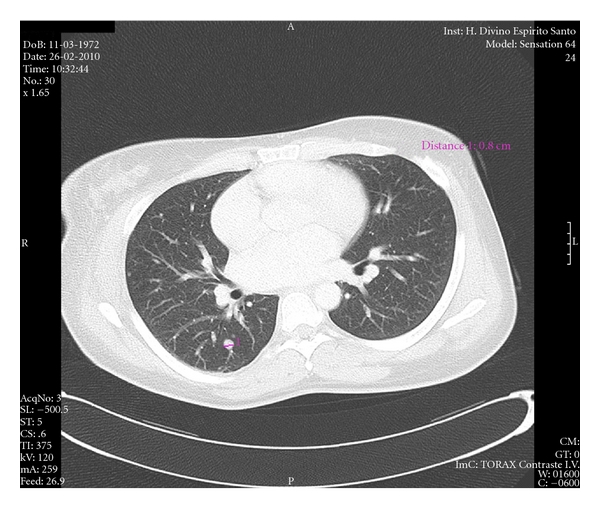
Thoracic CT scan. Multiple micronodules are visualized in peripheral topography, more numerous at the right lung, where an 8 mm nodule can be identified in the apical segment of the inferior lobe, with topography and morphology suggestive of metastases.

A thoracic contrast-enhanced CT scan showed multiple infracentimeric pulmonary metastases and thrombus progression had reached the right atrium (Figure 2). 

Despite chemotherapy with doxorubicin and iphosphamide, the disease progressed fatally over a few weeks.

A postmortem was not felt of value by the attending physicians, and thus was not performed.

## 3. Discussion

Leiomyosarcomas represent 10% to 15% of connective tissue sarcomas, 45% of retroperitoneal tumours [9], and 0.5% of sarcomas in adults [6]. A female gender preference has been reported, with a 6 to 1 ratio between female and male genders [10]. Primary venous leiomyosarcoma of the IVC is more frequent in women in their sixth decade [11] although reports of younger patients are found [12]. When the primary site of involvement is the lower limb, no gender preference is found [13].

Primary venous leiomyosarcomas arise from vascular smooth-muscle cells [7] and growth pattern may progress from intramural to endoluminal, extraluminal, or mixed forms [11]. Pathological classification follows retroperitoneal leiomyosarcoma criteria based upon mitotic figures count [14]. 

Approximately half of primary venous leiomyosarcomas originate in the IVC [4]. Less frequently, great saphenous vein (25%), femoral vein, internal jugular vein, and iliac vein involvement has been reported [7].

Clinical presentation of primary venous leiomyosarcomas is frequently oligosymptomatic and insidious [14] given their variable location allied to a slow growth rate, and its diagnosis may result of an incidental finding. However, at time of diagnosis, more than one half of the cases already present with pulmonary metastases [13]. Acute venous thrombosis is the most common presentation of the iliac primary venous leiomyosarcoma. Other symptoms may occur due to compression of the iliac artery and pelvic organs. Primary venous leiomyosarcomas are clinically divided into nonocclusive, occlusive or terminal [15]. Other frequent clinical presentations include abdominal pain [16], consumptive symptoms (loss of weight, anorexia, or asthenia), fever, night sweats, nausea, vomiting, and dyspnoea [17]. On physical examination an abdominal mass may be detected. Other less frequent presentations include haematuria [18], a renal mass [19], or a cardiac tumour [10].

Classically, the IVC is divided into 3 segments: segment I—the infrarenal IVC, segment III—proximal to the suprahepatic veins, and segment II—between the renal and suprahepatic veins [20]. Variable frequencies of segment involvement are featured among the literature, one series reporting segment I, II, and III involvement in 34%, 41,7%, and 24,3% respectively [21].

 Hematogenous spread commonly leads to secondary deposits in the lung, liver, and brain in primary venous leiomyosarcoma. In later stages, lymphatic pathways may be involved in metastization [22].

Occult tumour screening is indicated in recurrent or refractory to anticoagulation DVTs and may be considered if testing for other “benign” hypercoagulable conditions is inconclusive [23]. Diagnostic inaccuracy of complementary imaging has been reported in the literature concerning primary venous leiomyosarcoma [23] despite reports of high diagnostic sensibility (78% and 95%) and specificity (96% and 100%) of CT and MRI, respectively, for IVC thrombosis [10]. 

 Differential diagnosis includes retroperitoneal leiomyosarcomas, intravenous leiomyomatosis, endometrial stromal sarcoma, sarcomatoid renal cell carcinoma, angiosarcoma [4], and hemangioendothelioma [24].

Some authors have reported a high incidence (21%) of prior operated uterine tumours among patients with primary venous leiomyosarcoma of the IVC, suggesting an association. However, differential can be established through oestrogen and progesterone receptor testing [6].

Optimal treatment is yet to be established due to Primary Venous Leiomyosarcoma's limiting low incidence and consequent clinical experience [25]. However, an aggressive surgical approach assuring a free margin *en bloc* tumour resection can be curative [25] despite reported local recurrence rates in selected cases of 40 to 60% [10].

First-line chemotherapy with anthracycline derivates [26] and radiotherapy have failed to demonstrate long-term benefit in survival [6].

Poor prognostic factors include IVC segment III involvement, IVC occlusion, lower limb oedema, Budd-Chiari syndrome, intraluminal tumour growth [21], and diagnosis in advanced stages [14]. Patients with primary venous leiomyosarcoma have a mean survival of 3.5 years, and favourable outcome relies greatly upon eligibility and achievement of a free margin resection [5]. Comparatively to other sarcomas, primary venous leiomyosarcoma has a shorter metastases free interval and survival [26].

## 4. Conclusion

In regard to DVT, differential diagnostic considerations prior to thrombolytic treatment and surgery should include tumours, particularly if thrombolytic treatment proves ineffective. 

In our patient, CT and MRI played a controversial role, delaying the definitive diagnosis which was only achieved by a biopsy. These imaging modalities were inconclusive at an advanced stage of disease, and should any of these had been performed upon initial clinical presentation, 5 months before the definitive diagnosis, an even lower diagnostic sensibility might be expected.

Outcome depends upon tumour stage at diagnosis. Therefore, achieving a histopathologic diagnosis, in the presence of high clinical suspicion, is of the outmost importance in an effort to obtain an early-staged definitive diagnosis.

In conclusion, primary venous leiomyosarcoma is a rare cause of DVT and should be considered in recurrent or refractory DVT in young patients.
